# Persistent Wrist Pain

**Published:** 2015-07-28

**Authors:** Saptarshi Biswas

**Affiliations:** Department of Trauma and Acute Care Surgery, Allegheny Health Network, PA, USA

**Keywords:** osteoarthritis, pancarpal arthritis, proximal row carpectomy, wrist joint arthrodesis, wrist fusion

**Figure:**
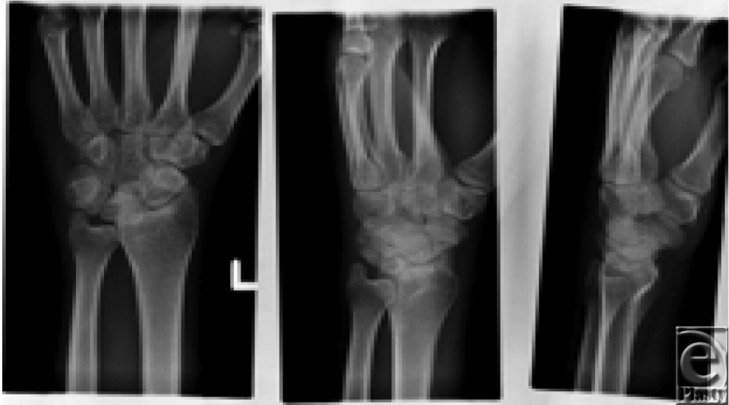


## DESCRIPTION

A 48-year-old manual laborer presented with persistent, severe painful wrist progressively getting worse. He was actively involved in sports for more than 35 years, which often resulted in him landing on his wrists repeatedly on hard grounds. His radiographs showed arthritic changes involving the wrist joints and carpal bones. His tests were negative for any autoimmune diseases.

## QUESTIONS

1. **What is the diagnosis?**
2. **What are the common causes?**
3. **What are the different known patterns of osteoarthritis (OA) of the wrist?**
4. **What are the treatment options?**

## DISCUSSION

This is a case of pan-OA of the wrist joint.

The common causes are OA and rheumatoid arthritis. Osteoarthritis can develop due to normal “wear and tear” in the wrist, particularly with a significant family history of arthritis. It may also develop as a result of a traumatic injury, such as fractured wrist bone or a wrist sprain. Scapholunate advanced collapse accounts for a significant number (∼55%) of degenerative wrist arthritis. It is a condition of progressive instability causing advanced arthritis of the radiocarpal and midcarpal joints. Wrist OA can also develop from Kienböck's disease where the blood supply to one of the small bones of the hand near the wrist (lunate) is interrupted resulting in death, which over time can lead to OA. The exact cause of rheumatoid arthritis is unknown. There may be a genetic predisposition. However, a chemical or environmental “trigger” might be required to activate the disease in those who genetically inherit rheumatoid arthritis.^1^^,^^2^

Wrist OA usually involves 4 patterns that determine the treatment option for each. (*a*) *Scaphoid, trapezoid, and trapezium (STT) joint arthritis*: Arthritis develops in the joint at the base of the thumb between the 3 carpal bones. (*b*) *Scapholunate advanced collapse pattern of arthritis*: Chronic scapholunate ligament injury causes scaphoid flexion and lunate extension resulting in abnormal force distribution across the midcarpal and radiocarpal joints. Radioscaphoid joint is affected first, followed by capitolunate joint. (*c*) *Scaphoid nonunion advanced collapse pattern of arthritis*: An unhealed scaphoid fracture may alter the biomechanics of the wrist joint resulting in “scaphoid nonunion advanced collapse.” (*d*) *Pancarpal arthritis***:** Arthritis involving majority of the wrist joint.^3^

Initial management is conservative consisting of analgesics, anti-inflammatory medication, and modification of activity. It is aimed at easing the pain and regaining motion. Surgery is considered when conservative measures fail.^3^^,^^4^ The type of surgery depends on the nature and longevity of the symptoms, type and extent of arthritis, and age and functional demands of the patient.

a. Surgery for STT arthritis
  i. *Excision of distal scaphoid with or without replacement*: Usually indicated for isolated STT arthritis. The far one third of the scaphoid bone is excised, and the gap may be filled by a tendon or interposed by a synthetic replacement.
  ii. *Fusion of STT joint*: In the young, fusion/arthrodesis of the joint may be considered that will provide a stable thumb for high-power manual work.
b. Surgery for scapholunate advanced collapse or scaphoid nonunion advanced collapse pattern of arthritis
  i. *Radial styloidectomy***:** In early stages of this pattern, excision of the styloid is recommended.
  ii. *Proximal row carpectomy*: Three carpal bones—scaphoid, lunate, and triquetrum—are excised and a new joint is created between the capitate and the radius, resulting in some movement of the wrist.^3^^,^^5^^,^^6^
  iii. *Scaphoid excision and 4-corner fusion*: The scaphoid is excised and the joints between the lunate, triquetrum, capitate, and hamate are fused. Approximately 50% to 60% of motion will be preserved.^3^^,^^7^
c. Surgery for pancarpal arthritis
  i. *Total wrist fusion*: The procedure is performed in young patients who have arthritis in one wrist and high physical demands on the wrist. The operation will lead to good pain relief but loss of movement of the wrist.^3^^,^^8^
  ii. *Total wrist replacement:* Performed in patients with arthritis in both wrists but with relatively low manual demands. The operation results in good pain relief and a functional range of movement.
  iii. *Wrist denervation*: In this procedure, nerve branches that take sensations from the wrist to the brain are cut, which will reduce pain perception and improve symptoms temporarily.
